# Polypharmacy and Potentially Inappropriate Medication in Iranian People With Metabolic Syndrome: Epidemiological Aspects and Related Factors, a Multi‐Level Cross‐Sectional National Study

**DOI:** 10.1002/hsr2.70600

**Published:** 2025-04-09

**Authors:** Mojdeh Daneshmand, Hamidreza Jamshidi, Mohammad‐Reza Malekpour, Erfan Ghasemi, Sahar Saeedi Moghaddam, Seyede Salehe Mortazavi, Mohsen Shati, Mohammad Hadi Farjoo, Farshad Farzadfar

**Affiliations:** ^1^ Department of Pharmacology, School of Medicine Shahid Beheshti University of Medical Sciences Tehran Iran; ^2^ Non‐Communicable Diseases Research Center, Endocrinology and Metabolism Population Sciences Institute Tehran University of Medical Sciences Tehran Iran; ^3^ Non‐Communicable Diseases Research Center Tehran University of Medical Sciences Tehran Iran; ^4^ Geriatric Mental Health Research Canter School of Behavioural Sciences and Mental Health Iran University of Medical Sciences Tehran Iran; ^5^ Department of Epidemiology, Psychosocial Health Research Institute Mental Health Research Centre Tehran Iran

**Keywords:** metabolic syndrome, polypharmacy, potentially inappropriate medication, sociodemographic

## Abstract

**Background and Aims:**

Polypharmacy, characterized by the concurrent use of five or more medications in a prescription, potentially resulting in adverse outcomes, is frequently observed among individuals with metabolic syndrome, which encompasses a collection of conditions that co‐occur, heightening the likelihood of heart disease, stroke, and type 2 diabetes. This study seeks to ascertain the prevalence of polypharmacy and the use of potentially inappropriate medications (PIMs) among Iranian patients with metabolic syndrome, while also evaluating the contributing individual and sociodemographic factors.

**Methods:**

This was a population‐based, cross‐sectional national study. Two databases were used: (a) Iranians Health Insurance Service database and (b) Iran's STEPS 2016 survey. Patients with metabolic syndrome conjoint in both databases were selected. Among these patients, polypharmacy and PIM were evaluated, and their association with individual and sociodemographic factors was assessed. Univariate and multivariate logistic regression were used to analyze the associations. All statistical analyses were done using SPSS 22 and Python 3.

**Results:**

Out of 2075 metabolic syndrome patients, 10.3% had polypharmacy. Polypharmacy significantly increased by age (OR: 4.334, adjusted for > 80‐year‐olds vs. 25–39‐year‐olds [CI: 1.664–11.283], *p* < 0.001), and its prevalence was significantly higher in urban areas (OR: 2.326 [CI: 1.645–3.288], *p* < 0.001). Polypharmacy was 5.88% in West, 5.41% in Southeast, 5.04% in Central, and 4.83% in North‐Northeast of Iran. PIM was 13.2% in ≥ 60 years and significantly higher in urban areas (OR: 2.014 [CI: 1.153–3.519], *p* < 0.001).

**Conclusions:**

Since the area of residency affects polypharmacy and PIM more significantly than wealth status and education level, it is important to implement preventive measures in urban areas.

## 1 Introduction

Polypharmacy, a term coined more than a century ago, describes the utilization of multiple drug regimens. According to the references, there are different definitions of polypharmacy, mostly defining it as the concurrent use of ≥ 5 drugs regardless of clinical indication [1, 2, 3]. Polypharmacy can stem from both physicians and patients and is influenced by factors like communication gaps, misdiagnosis, improper prescriptions, and inadequate coordination of physicians [4]. Patients may also contribute through self‐medication or obtaining multiple prescriptions from different healthcare providers [5, 6]. Moreover, according to different studies, sociodemographic factors such as age, education level, and lifestyle can also affect polypharmacy [7]. Although polypharmacy is not always harmful, it can heighten the likelihood of drug interactions, diminish the quality of life, increase morbidity and mortality rates, and incur expenses for both patients and the healthcare system [8].

In older adults, the likelihood of drug intolerance and medication errors increases due to a higher susceptibility to polypharmacy stemming from multiple comorbidities and often poor adherence, which could lead to adverse drug reactions [9, 10, 11, 12, 13]. Potentially inappropriate medication (PIM) is one of the major risk factors for adverse drug events [14, 15]. Therefore, it is crucial to assess the possibility of PIM in the elderly as a consequence of polypharmacy [15]. According to studies, functional status, depression, health quality, economic situation, a high comorbidity score, reduced cognition, self‐medication, and high risk of clinical‐functional vulnerability are some of the confounding factors for PIM [16]. Since these factors may be different in various societies, it is important to assess these covariates in Iran as well.

Among various diseases, metabolic syndrome is a constellation of different risk factors associated with some cardiovascular diseases and diabetes mellitus and is growing worldwide. Due to MetS's various comorbidities, patients often face polypharmacy to manage their complications [17, 18, 19]. Also, according to different studies, polypharmacy is associated with lower therapeutic benefits and higher rates of morbidity and mortality [20, 21, 22]. Some studies have also found that with multiple therapies, the cost–benefit ratio drops in chronic diseases [23, 24]. Therefore, it is important to assess the prevalence and confounding factors of polypharmacy among MetS patients. This study examined polypharmacy and PIM in the prescriptions of individuals with MetS, alongside their correlation with both individual and sociodemographic factors, which, to our knowledge, has not been conducted at this scale in Iran before.

## 2 Methods

### 2.1 Dataset

This study was a cross‐sectional, multi‐level national investigation utilizing data from the Iranian Health Insurance and the STEPS Iran 2016 survey. The STEPS survey employed in this study is an ongoing, sequential, large‐scale, cross‐sectional population‐based surveillance of four noncommunicable diseases (cardiovascular, diabetes, respiratory diseases, and cancer) across all 31 provinces of Iran at the national level. Approximately 31,050 individuals aged 25 years and above were randomly enrolled in the STEPS 2016 study and identified by their Unique National ID. Their demographic and epidemiological information, as well as risky behaviors, were documented through laboratory data, medical examinations, and questionnaires [25]. To identify patients with available prescription data as well as individual and sociodemographic information, we compared the national ID of patients from the 102 million prescriptions recorded in the Iranian Health Insurance dataset for 2015 and 2016 with the national ID of patients included in the STEPS 2016 survey dataset in Iran. Approximately 16,000 patients were chosen based on this overlap. Subsequently, we categorized the prescriptions of these patients, along with their medications, using Anatomical Therapeutic Chemical (ATC) codes [26]. Among these 16,000 patients, MetS patients were separated based on the American Heart Association criteria [27, 28].

To identify prescriptions with polypharmacy, we followed these steps: (a) one prescription for each MetS patient was randomly selected from the Iranian Health Insurance dataset for each year (2015 and 2016). (b) Given the chronic nature of MetS, we assessed whether medications recurred in patients' other prescriptions within the same year, including only drugs repeated at least three times annually [29]. (c) If the aforementioned criterion was met, we tallied the number of medications in each specific prescription to evaluate polypharmacy, defined as five or more medications within a single prescription.

### 2.2 Data Classification

**Figure 1:**
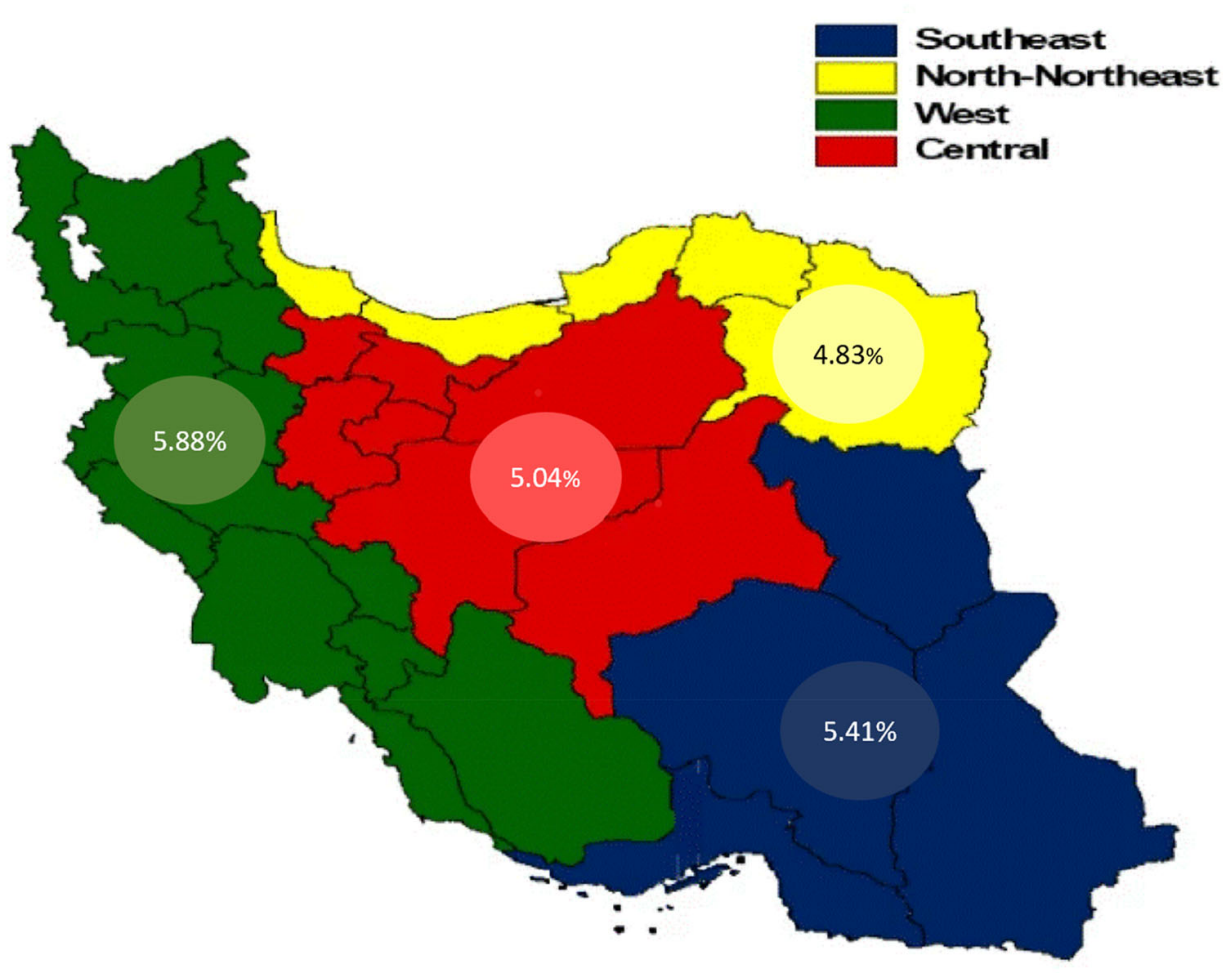
Distribution of polypharmacy as % in four regions of Iran. Courtesy of Farzadfar et al. [31].

The study examined traits of MetS patients, including gender, residential area, age, marital status, and education level. Additionally, it utilized a wealth index as outlined in the STEPS protocol to categorize patients into five economic groups [30]. The populations of provinces were age‐standardized (except Qom, since it was not included in the STEPS study), and polypharmacy prevalence was assessed across four regions in Iran, based on a previous study, which divided the country into four regions of North‐Northeast, West, Central, and Southeast based on geography and socioeconomic state (SES) of the patients [31] (Figure 1). To mitigate PIM risks, the American Geriatrics Society (AGS) issues a list of medications unsuitable for adults aged 65 and above, based on the Beers criteria [32]. However, as many developing countries, including Iran, define elderly age starting from 60, we adopted this threshold [32, 33, 34]. Beers criteria provide a list of drugs to be avoided in the elderly, categorized based on low, moderate, and high quality of evidence. In this study, we only chose the drugs in the moderate and high categories as PIM. Logistic regression analyses were performed by merging data from 2015 to 2016, considering any prescription with polypharmacy or PIM in either year.

### 2.3 Statistical Analysis

In this study, we aimed to evaluate the effect of individual and sociodemographic factors on polypharmacy and PIM. To do so, descriptive statistical analysis was done based on both individual factors (gender and age) and sociodemographic factors (education level, area of living, marital status, and wealth index) as independent variables. First, polypharmacy was considered as a dependent variable, and individual and sociodemographic factors were all considered independent variables. Afterward, drugs considered PIMs were defined based on the Beers criteria, and then using this data and prescription data among patients 60 years and above, python programming was used to separate these patients into two groups with and without PIM. Subsequently, PIM was considered as the dependent variable, and polypharmacy, individual, and sociodemographic factors were all considered as independent variables. Crude (univariate) and adjusted logistic regression (multivariate) models were used to assess the associations. SPSS version 22 and Python programming system version 3 were used for data analyses with a significance level set at *p* ≤ 0.05.

### 2.4 Ethics Approval

The Ethical Committee of Shahid Beheshti University of Medical Sciences approved the study of the Medical School Pharmacology under reference number IR.SBMU.MSP.REC.1400.004.

## 3 Results

### 3.1 General Information

MetS patients totaled 2075 (12.9%), with 1391 (67.03%) females, having a mean (SD) age of 54.75‌‌ (14). The prevalence of polypharmacy was 10.3%, escalating from 2.8% in the 25–39 age group to 17.2% in the 60–80 age bracket before declining to 12.9% in those over 80 years old (*p* < 0.001) (Table 1).

**Table 1:** Individual and sociodemographic characteristics of MetS patients based on polypharmacy (data of patients with no tendency to answer are not shown).

Factor	Total no. (%)	Polypharmacy (%)	Crude OR^c^	*p* value (crude OR)	CI (95%) (crude OR)	Adjusted OR	*p* value (adjusted OR)	CI^d^ (95%) (adjusted OR)
Gender								
Female	1391 (67.03)	139 (10)						
Male	684 (32.96)	75 (11)	1.11	0.5	0.824–1.493	1.05	0.8	0.758–1.457
Age group				< 0.001*			< 0.001*	
25–39	316 (15.2)	9 (2.8)						
40–59	993 (47.9)	77 (7.8)	2.87	0.003*	1.420–5.788	2.50	0.01*	1.226–5.103
60–80	681 (32.8)	117 (17.2)	7.08	< 0.001*	3.542–14.137	6.31	< 0.001*	3.106–12.815
> 80	85 (4.1)	11 (12.9)	5.07	0.001*	2.027–12.683	4.33	0.003 *	1.664–11.283
Area of living								
Rural	761 (36.7)	48 (6.3)						
Urban	1314 (63.3)	166 (12.6)	2.15	< 0.001*	1.537–3.001	2.33	< 0.001*	1.645–3.288
Years of schooling				0.7			0.7	
0	597 (28.8)	57 (9.5)						
1–6	650 (31.3)	68 (10.5)	1.11	0.6	0.764–1.604	1.15	0.5	0.783–1.678
7–12	527 (25.4)	60 (11.4)	1.22	0.3	0.830–1.785	1.21	0.3	0.815–1.799
> 12	301 (14.5)	29 (9.6)	1.01	0.9	0.631–1.616	0.94	0.8	0.580–1.539
Marital status^a^				0.01*			0.5	
Never married	55 (2.7)	1 (1.8)						
Married	1722 (83)	170 (9.9)	5.91	0.08	0.813–43.027	3.15	0.3	0.422–23.881
Divorced/separated	48 (2.3)	4 (8.3)	4.91	0.2	0.529–45.525	2.99	0.3	0.310–28.759
Widow	244 (11.8)	38 (15.6)	9.96	0.03*	1.337–74.200	3.9	0.2	0.499–30.493
Wealth Index^b^				0.2			0.3	
Poorest	463 (22.3)	51 (11)						
Poor	434 (20.9)	52 (12)	1.1	0.6	0.729–1.658	1.23	0.3	0.809–1.886
Average	408 (19.7)	46 (11.3)	1.03	0.9	0.673–1.567	1.08	0.7	0.695–1.668
Rich	372 (17.9)	36 (9.7)	0.87	0.5	0.552–1.358	0.91	0.7	0.571–1.437
Richest	377 (18.2)	28 (7.4)	0.65	0.08	0.400–1.050	0.72	0.2	0.440–1.180
Total	2075 (12.9%)	214 (10.3)						

* Indicates the significance of the association.

^a^ 0.3% of patients did not give an answer based on their marital status.

^b^ 1% of patients had no tendency to answer for their wealth index.

^c^ OR: odds ratio.

^d^ CI: confidence interval.

Of the total, 1314 (63.3%) patients resided in urban areas. Polypharmacy was 6.3% in rural areas and 12.6% in urban settings (OR: 2.33 [CI: 1.645–3.288], *p* < 0.001) (Table 1).

### 3.2 Regional Distribution of Polypharmacy

Polypharmacy exhibited the highest prevalence in the Western regions of Iran, while the lowest prevalence was observed in the North‐Northeastern areas (Figure 1).

### 3.3 PIM

In 2015 and 2016, among 766 patients aged 60 years and above, 13.2% were identified with PIM. The prevalence of PIM was 6.6% among patients without polypharmacy, contrasting with 46.1% among those with polypharmacy (OR: 11.6 [7.154–19.023], *p* < 0.001). PIM occurrence was notably higher in urban settings (16.8%) (OR: 2.01, [CI: 1.153–3.519], *p* = 0.01) (Table 2).

**Table 2:** Individual and sociodemographic characteristics of patients above 60 years (data of patients with no tendency to answer are not shown).

Factor	No. (%)	PIM no.^b^ (%)	*p* value	Adjusted OR^a^	CI^c^ (95%)
Total	766 (36.9)	101 (13.2)			
Age group					
60–80	699 (91.3)	92 (13.5)			
> 80	67 (8.7)	9 (10.6)	0.7	0.83	0.365–1.896
Gender					
Female	496 (64.8)	66 (13.3)			
Male	270 (35.2)	35 (13.0)	0.4	0.8	0.483–1.337
Area of living					
Rural	295 (38.5)	22 (7.5)			
Urban	471 (61.5)	79 (16.8)	0.01*	2.01	1.153–3.519
Years of schooling					
0	389 (50.8)	31 (14.0)			
1–6	189 (24.7)	31 (13.7)	0.9	0.95	0.519–1.764
7–12	105 (13.7)	31 (15.3)	0.7	1.11	0.601–2.067
> 12	83 (10.8)	8 (6.8)	0.03	0.36	0.140–0.912
Wealth Index					
Poorest	196 (25.6)	28 (14.3)			
Poor	149 (19.5)	21 (14.1)	0.9	0.98	0.488–1.980
Average	153 (20.0)	16 (10.5)	0.1	0.58	0.276–1.215
Rich	138 (18.0)	20 (14.5)	0.6	1.23	0.604–2.518
Richest	121 (15.8)	15 (12.4)	0.9	0.97	0.450–2.095
Polypharmacy					
No	638 (83.3)	42 (6.6)			
Yes	128 (16.7)	59 (46.1)	< 0.001*	11.6	7.154–19.023

* Indicates the significance of the association.

^a^ OR: odds ratio.

^b^PIM: potentially inappropriate medication.

^c^ CI: confidence interval.

## 4 Discussion

In this study, polypharmacy exhibited a significant correlation with age and was notably higher in urban regions of Iran, affecting 10.3% of patients in 2015 and 2016. This prevalence was reported as 7%–45% based on an umbrella study, which assessed 11 systematic reviews of different countries [35]. A systematic review and meta‐analysis on polypharmacy in Europe, Asia, North America, Australia, South America, and Africa, among 54 studies, reported 37% polypharmacy, which showed that sex and geographical location were not associated with differences in polypharmacy prevalence [36]. In another study in one of the provinces of Iran, the prevalence of polypharmacy was 9.51% among over 14,000 individuals, which is closer to our results [37]. Although all these studies considered polypharmacy as the concomitant use of 5 or more medications, none of them used our criterion that considered only the drugs repeated 3 times or more in the prescriptions of 1 year. This may be the reason for our lower prevalence, but it gives us insight into the chronic use of medications.

Among the elderly, the prevalence of PIM was 13.2%. Compared with the United Kingdom and Canada, the prevalence was notably lower in Iran. The United Kingdom exhibited a higher prevalence at 31% [38], while Canada reported an even higher rate of 56% among older adults with diabetes [39]. These differences in findings may stem from our focus solely on MetS patients as our target population, as well as our stringent criteria for defining polypharmacy, which required medications to be repeated three times or more each year.

Polypharmacy escalates significantly with age in Iran, reaching its peak in urban locales. However, there is a decrease in patients aged over 80 compared to those in the 60–80 age bracket, potentially attributed to the latter group having a higher burden of comorbidities. This trend mirrors findings from a study conducted on elderly Italians, where polypharmacy prevalence was higher among older individuals and urban residents [40]. Enhanced access to medical facilities and healthcare providers in urban areas of Iran likely contributes to the observed higher rates of polypharmacy. Conversely, the rural regions of Iran operate under a referral system wherein patients are initially assessed by a family physician and then, if necessary, referred to a specialist. This systematic approach may serve as a preventive measure against patients receiving multiple prescriptions.

The richest patient group exhibited the lowest prevalence of polypharmacy. While this association lacked significance, it challenges the notion, at least within Iran, that higher‐income individuals consume more medications. This finding may stem from the inclination and capacity of wealthier patients to consult specialist physicians, potentially mitigating the risk of unnecessary polypharmacy. Similarly, a cross‐sectional study involving 730 patients in England found that polypharmacy was notably more prevalent in lower‐income groups [41].

Regionally, the prevalence of polypharmacy was highest in the West, and lowest in the North‐Northeast of Iran. Although the Central region of Iran has the highest SES [31], polypharmacy was rather lower in this region. This finding again confirms the idea that higher SES does not necessarily increase polypharmacy.

A significant association of PIM with polypharmacy and urban residence suggests a potential need for closer medication management among the elderly with multiple prescriptions and those residing in urban areas. In the current study, no significant association was observed between PIM, education level, and wealth index; in contrast, in Sweden, lower education levels among women were linked to higher rates of both polypharmacy and PIM, emphasizing the importance of education in medication management [1]. In France, sociodemographic status played a significant role, with unemployment and lower household incomes correlating with increased PIM prevalence [42]. Similarly, in the United States, lower education and income levels were associated with higher PIM prevalence, indicating a need for targeted interventions to address healthcare disparities among socioeconomically disadvantaged populations [43]. These differences highlight the importance of the assessed population, considering local context, cultural factors, urbanization, education, socioeconomic status, geographical differences, as well as healthcare system characteristics, and regulations governing physician prescription standards. Addressing these factors through comprehensive healthcare policies and interventions could help reduce the burden of inappropriate medication use among patients and, especially, older adults.

### 4.1 Strengths

This study was performed on an individual level based on an original database, on a national scale. The definitions were considered strictly, and the study was solely done on MetS patients, as they are prone to polypharmacy due to multiple complications. In polypharmacy assessment, we only counted medications that appeared in prescriptions at least three times over the course of a year. This approach provides a more accurate understanding of polypharmacy among MetS patients.

### 4.2 Limitations

This was a cross‐sectional study; therefore, further studies are required to assess the trend of the issue over periods of time. The data used in this study was a subset of the STEPS survey dataset; therefore, the data related to the provinces was age‐standardized and combined in four large regions of Iran to gain more reliable statistical results. According to the STEPS survey, Qom province did not provide data for analyses, which might alter the results. Moreover, in addition to prescription drugs, over‐the‐counter drugs are also obtained by metabolic syndrome patients; therefore, this compromises the actual number of polypharmacy and PIM.

## 5 Conclusion

These results highlight the importance of implementing measures to control unnecessary polypharmacy, thus mitigating the risk of PIM and medication errors. For example, leveraging the referral system in rural areas of Iran could serve as a beneficial strategy to reduce polypharmacy in urban settings; furthermore, enhancing electronic prescribing systems in Iran could aid in detecting prescription errors and unnecessary polypharmacy by both prescribers and pharmacists. Future research endeavors could focus on analyzing more recent datasets to discern trends in polypharmacy over time. Additionally, exploring diverse insurance databases and examining prescriptions without insurance coverage could provide valuable insights into variations in polypharmacy patterns.

## Author Contributions

**Mojdeh Daneshmand:** investigation, writing – original draft, visualization, writing – review and editing, formal analysis, data curation. **Hamidreza Jamshidi:** conceptualization, validation, project administration, supervision. **Mohammad‐Reza Malekpour:** investigation, formal analysis, software. **Erfan Ghasemi:** software, formal analysis. **Sahar Saeedi Moghaddam:** formal analysis. **Seyede Salehe Mortazavi:** supervision, validation. **Mohsen Shati:** validation, supervision. **Mohammad Hadi Farjoo:** investigation, writing – review and editing, supervision. **Farshad Farzadfar:** conceptualization, methodology, validation, project administration, data curation, supervision, resources.

## Ethics Statement

The Ethical Committee of Shahid Beheshti University of Medical Sciences approved the study of the Medical School Pharmacologyunder reference number IR.SBMU.MSP.REC.1400.004.

## Consent

The data used for this study is derived from the STEPS Iran 2016 survey, and the aforementioned consent has already been obtained by the survey authorities.

## Conflicts of Interest

The authors declare no conflicts of interest.

### 1 Transparency Statement

Dr. Mojdeh Daneshmand; the lead author, affirms that this manuscript is an honest, accurate, and transparent account of the study being reported; that no important aspects of the study have been omitted; and that any discrepancies from the study as planned (and, if relevant, registered) have been explained.

## Data Availability

The data that support the findings of this study are available on request from the corresponding author. The data are not publicly available due to privacy or ethical restrictions. The datasets generated during and/or analyzed during the current study are not publicly available due to confidentiality but are available from the corresponding authors upon reasonable request.
